# Challenges in updating habitat suitability models: An example with the lesser prairie-chicken

**DOI:** 10.1371/journal.pone.0256633

**Published:** 2021-09-20

**Authors:** Catherine S. Jarnevich, Pairsa N. Belamaric, Kent Fricke, Mike Houts, Liza Rossi, Grant Beauprez, Brett Cooper, Russell Martin

**Affiliations:** 1 U.S. Geological Survey, Fort Collins Science Center, Fort Collins, Colorado, United States of America; 2 Student contractor to the U.S. Geological Survey, Fort Collins Science Center, Fort Collins, Colorado, United States of America; 3 Kansas Department of Wildlife, Parks and Tourism, Emporia, Kansas, United States of America; 4 Kansas Biological Survey, University of Kansas, Lawrence, Kansas, United States of America; 5 Colorado Parks and Wildlife, Steamboat Springs, Colorado, United States of America; 6 New Mexico Department of Game and Fish, Texico, New Mexico, United States of America; 7 Oklahoma Department of Wildlife Conservation, Woodward, Oklahoma, United States of America; 8 Texas Parks and Wildlife Department, Canyon, Texas, United States of America; Southeastern Louisiana University, UNITED STATES

## Abstract

Habitat loss from land-use change is one of the top causes of declines in wildlife species of concern. As such, it is critical to assess and reassess habitat suitability as land cover and anthropogenic features change for both monitoring and developing current information to inform management decisions. However, there are obstacles that must be overcome to develop consistent assessments through time. A range-wide lek habitat suitability model for the lesser prairie-chicken (*Tympanuchus pallidicinctus*), currently under review by the U. S. Fish and Wildlife Service for potential listing under the Endangered Species Act, was published in 2016. This model was based on lek data from 2002 to 2012, land cover data ranging from 2001 to 2013, and anthropogenic features from circa 2011, and has been used to help guide lesser prairie-chicken management and anthropogenic development actions. We created a second iteration model based on new lek surveys (2015 to 2019) and updated predictors (2016 land cover and cleaned/updated anthropogenic data) to evaluate changes in lek suitability and to quantify current range-wide habitat suitability. Only three of 11 predictor variables were directly comparable between the iterations, making it difficult to directly assess what predicted changes resulted from changes in model inputs versus actual landscape change. The second iteration model showed a similar positive relationship with land cover and negative relationship with anthropogenic features to the first iteration, but exhibited more variation among candidate models. Range-wide, more suitable habitat was predicted in the second iteration. The Shinnery Oak Ecoregion, however, exhibited a loss in predicted suitable habitat that could be due to predictor source changes. Iterated models such as this are important to ensure current information is being used in conservation and development decisions.

## Introduction

Species distribution models are often used to support conservation decisions [1,2], including prioritizing locations and actions for conservation and management. Sofaer et al. [1] identified iterative modeling as one of several criteria in an assessment rubric to evaluate and communicate species distribution model use and quality. Most species distribution models are static, representing a single point in time. Without regular updates, predictions can become out-of-date and thus less accurate [3]. Iterative models can incorporate new location and predictor data to produce updated maps of habitat suitability [e.g., 4, 5]. The routine iteration of these models could provide valuable information regarding a species’ habitat associations and current geographical location for managers and biologists. For example, the U.S. Department of Agriculture gypsy moth surveillance program is guided by models that are iterated annually, incorporating both new observation data and up-to-date predictors [4]. Iterative modeling may also serve as a powerful tool to inform the conservation and protection of threatened or endangered species as habitat changes through time. This ideally would involve a relatively automated process whereby new survey data and up-to-date predictors are included as they become available to update prediction [6]. Comparing iterative models can prove challenging, however, as predicted changes between model iterations could reflect on-the-ground habitat changes or changes in methods or inputs. While climate data are systematically updated over time, such as the weather station data used in White et al. [6], this is not common for many other data sets.

The lesser prairie-chicken (*Tympanuchus pallidicinctus*) is a prairie grouse species of conservation concern, whose range encompasses the Southern Great Plains region of Colorado, Kansas, New Mexico, Oklahoma, and Texas [7,8]. Their display grounds, or leks, tend to be in elevated locations with minimal vegetation [9] that are in close proximity to densely vegetated, vertically structured nest (mid-height to tall grasses) [10] and brood (forbs subshrubs and shrubs) habitat [11]. Lesser prairie-chickens conduct most of their activities and complete their life cycle within 1500 m of known leks [12–14]. Landscape characteristics related to reproductive habitat within 5000 m of a lek can influence lek attendance [11]. Populations have fluctuated over time across the range [15], with population decline linked to habitat fragmentation, degradation, and loss resulting from energy production [16], agricultural conversion, anthropogenic features [16–19], and woody encroachment [20,21]. Landscape change continues to occur, as evidenced by land cover time series such as changes between different broad land cover classes [22] and increased energy development in the region, particularly New Mexico and Texas [23].

A group of modelers, wildlife biologists, and wildlife management agency personnel co-produced a lek habitat suitability model for the lesser prairie-chicken [24]. The model was based on lek location data from 2002–2012 and 11 predictors representing the ecological and anthropogenic features from a period circa 2011 (data dependent on predictor). Lek suitability was modeled because leks are considered a focal point for prairie grouse management [25], lek locations are surveyed annually using aerial and ground-based monitoring methods throughout the lesser prairie-chicken range, and breeding habitats, a primary determinant in lesser prairie chicken populations [26], are closely associated with lek sites [14,27]. The predictors included in the model were based on input from wildlife management agency personnel. However, in subsequent years, landscapes in the Southern Great Plains of the U.S. have changed and are projected to continue to change [28]. It is important to understand changes in habitat suitability over time to ensure management actions are based on the most recently available predictor data. To maintain up-to-date predictions of lek habitat suitability for lesser prairie-chickens across their range, it is critical to periodically update models with new lek occurrences and current landscape condition data sets as they become available. Our initial goal was to apply the first iteration model to temporally updated predictors to evaluate changes in suitable habitat. Nevertheless, as we will show, this goal proved difficult. We modified our goal to instead quantify changes in the amount of predicted suitable habitat based on a second iteration model. We highlight potential challenges that may arise with iterating models with the goal of identifying changes in suitability related to trade-offs in using the best available data and data that can easily be updated temporally. We provide updated habitat suitability predictions to agencies and habitat managers who are using the first iteration habitat suitability model in decision making and evaluate changes in important land use/land cover categories in the study region.

## Methods and materials

### Study area

**Fig 1:**
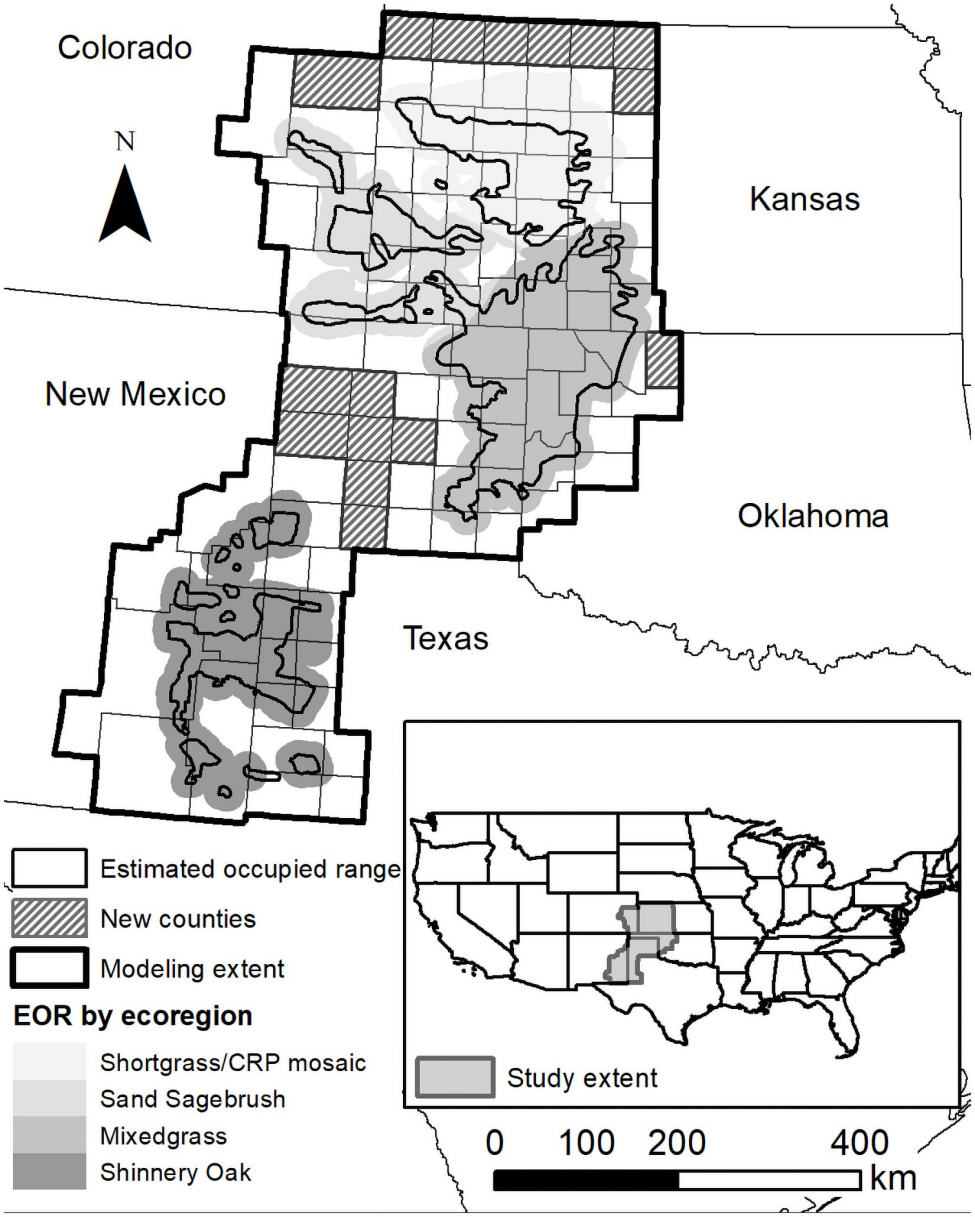
Study site location map. The extent of the second iteration habitat suitability model for lesser prairie-chicken leks across the five states of its current range, overlaid with the species’ Estimated Occupied Range (EOR) area, buffered by 16.1 km, and shaded by ecoregion. Second iteration study extent added the new counties to the initial iteration extent. [Projected Coordinate System: Albers Conic Equal Area].

This study, the development of a second iteration model, includes an expanded region, incorporating the 2011 estimated occupied range (EOR) of the lesser prairie-chicken buffered by 16.1 km (10 mi) as defined by the Lesser Prairie-Chicken Interstate Working Group and mapped on the Southern Great Plains Crucial Habitat Assessment Tool (https://www.sgpchat.org/; Fig 1). The EOR is a western association of fish and wildlife agencies lesser prairie chicken range-wide conservation plan delineation based on experts that is buffered by 10 miles to capture potential annual variability from shifting habitat, population size, and detectability. We expanded the study area to county boundaries that match political jurisdictions for management applications and added counties on the northern edge where new populations have been detected [29]. This resulted in a 32,868,102-ha area, covering 105 counties in Colorado, New Mexico, Kansas, Oklahoma, and Texas compared to the 89 counties (28,420,417-ha area) included in Jarnevich et al. [24]. Our expanded analysis area is inclusive of the four ecoregions that comprise the lesser prairie-chicken range [8]. Analyses focused on the study area, the entire EOR+10 (the buffered estimated occupied range), and each of the four ecoregions within the EOR+10.

### Predictor updates

State wildlife management agency representatives, in consultation with modelers and biologists, chose 11 predictors at a 210 m resolution for the first iteration model [Table 1; 24]. For the second iteration, we used updated versions of all the predictors except for the categorical U.S. state predictor, which distinguished the five different states covered by the study area, and topographic position index, which can highlight higher locations on the landscape that are used for lekking [30], as neither of these have changed since the first iteration model development (see Table 1 for exact dates by predictor).

**Table 1:** Methods and sources used to create predictor variables used to model lesser prairie-chicken lek suitability.

Predictor	Creation step	Modification	Range
Distance to FAA structures [1]	Euclidean distance (km) to FAA structures taller than 50 ft using ArcGIS Spatial Analyst tools	Created with FAA structure locations from 1973 to 2011 (first iteration) and 1973 to 2019 (second iteration).	0.03 to 31 km
Distance to highway [2]	Euclidean distance (km) to highways using ArcGIS Spatial Analyst tools	ESRI ArcGIS Data and Maps, version 10, “mroads” layer including Primary = S1100 or S1200 for both iterations; second iteration manual clean-up of misclassified road types.	0.02 to 32 km
Distance to roads [2]	Euclidean distance (km) to roadways using ArcGIS Spatial Analyst tools	ESRI ArcGIS Data and Maps, version 10, “mroads” layer including Secondary = S1400for both iterations; second iteration had manual clean-up of misclassified road types.	0.01 to 12 km
Distance to transmission line [3]	Euclidean distance (km) to transmission lines using ArcGIS Spatial Analyst tools	First iteration data purchased from Platts “North America Electric Transmission Lines GIS Layer,” in April 2011; second iteration data from new freely available data set observed through 2018.	0 to 48 km
Well density [4]	Filtered points to exclude wells with indication of inactive or plugged. Point statistics in ArcGIS Spatial Analyst neighborhood functions, summing the number of points in a circle with a 1600 m radius.	Well data compiled from each state government including spud dates of 1900 to 2011 (first iteration) and of 1900 to 2019 (second iteration).	0 to 48.24 wells
Average EVI [5]	Average of average annual Enhanced Vegetation Index (EVI) from 2008 to 2017	Original MODIS phenology product (MCD12Q2 v006) averaged across 2000 to 2009; updated was generated from raw MODIS data for the most recent decade available	0.04 to 0.41
Percent used land cover types within 5000 m [6, 7]	Classified shrubland (51, 52), grassland/herbaceous (71), and pasture/hay (81) as known/suspected used unless overlapping with woody encroachment. Focal Statistics (suit_*, Sum) / Focal Statistics (constant, Sum), Calculated for a 5000 m radius, then resampled to 210 m.	First iteration used the Southwest Regional Gap Land Cover Dataset (2001) in Colorado and New Mexico, the NLCD (2011) for Kansas and Oklahoma, and Ecological Mapping Systems of Texas (2013) with categories in each assigned to known/suspected used or unused categories and input into same focal statistics equation. Second iteration based on NLCD 2016, classified in same way as NLCD 2011, with woody encroached areas removed from known/suspected used.	0 to 100%
CRP within 5000 m [8]	Focal Statistics (suit_*, Sum) / Focal Statistics (constant, Sum) * 100, calculated for a 5000 m radius, then resampled to 210 m	CRP data from 2012 (first iteration) and 2019 (second iteration). Directly comparable.	0 to 70%
Land Condition Index [9]	Created from transportation corridors, urban and industrial development, and managed/modified land cover data in 2017 [31]. Scale was 0 to 1, with higher values representing less human impact.	Original landscape condition layer produced in 2012 had a different scale (1–10,000) and inputs [32].	0 to 1
State	Categorical value for each of the five states	Used original layer from Jarnevich et al. [24]	n/a
Topographic Ruggedness Index (TRI)	Topographic Ruggedness Index calculated following Riley et al. [33]; resampled to 210 m	Used original layer from Jarnevich et al. [24]	0 to 27

Predictor name, steps taken to create the predictor, changes in predictor from the first iteration predictor [24] (“modification”), and the range in values (minimum to maximum) in the training data including both presence and background locations for all predictors used to model lesser prairie-chicken lek suitability. FAA indicates Federal Aviation Administration, CRP refers to the Conservation Reserve Program, and EVI is enhanced vegetation index.

^1^ Digital Obstacle File (DOF): https://www.faa.gov/air_traffic/flight_info/aeronav/digital_products/dof/.

^2^ Roads layer from ESRI: https://www.arcgis.com/home/item.html?id=871852b13b53426dabdf875f80c04261.

^3^ Homeland Infrastructure Foundation-Level Data: https://hifld-geoplatform.opendata.arcgis.com/datasets/electric-power-transmission-lines.

^4^ Well data compiled for each state, removing those with indication no longer active or plugged: https://wellsdatabase.com.

^5^ MODerate resolution Imaging Spectroradiometer (MODIS) Vegetation Indices: https://lpdaac.usgs.gov/products/mod13q1v006/.

^6^ National Land Cover Dataset (NLCD) 2016: https://www.mrlc.gov/data/nlcd-2016-land-cover-conus.

^7^ Woody encroachment: http://pljv.org/for-habitat-partners/maps-and-data/data-downloads/.

^8^ Conservation Reserve Program (CRP) information summarized from 2019.

^9^ Comer and Hak (2017): https://www.natureserve.org/conservation-tools/modeling-landscape-condition.

In the first model iteration, potential land cover sources were evaluated by state wildlife management agency representatives, as some sources better represented different states within the study region. Land cover categories were based on the National Land Cover Dataset (NLCD) 2011 product for Kansas and Oklahoma; Colorado and New Mexico were based on Southwest Regional Gap Land Cover Dataset [34] and Texas was based on the Ecological Mapping Systems of Texas [35] land cover data set. Because these data sets had different vegetation mapping categories, each state’s representatives defined classes as those known/suspected to be used by lesser prairie-chickens (hereafter known/suspected used) based on research and expert knowledge to develop a compatible data set across the three data sources. These classes focused on grasslands (including pasture/hay) and shrublands as combinations of these broad classes are important for the species [e.g., 11]. Additionally, we were not interested in the specific land cover type at a location, but rather the composition of vegetation in the area around a location. In the first iteration, Jarnevich et al. [24] compared models using different radii based on utilized area around leks (~1600 m [12–14] to ~5000 m [11,20]) to calculate the percent of land cover types known/suspected used by lesser prairie-chickens within a specified radius neighborhood. Jarnevich et al. [24] selected a 5000 m radius and we used this same neighborhood size.

For the second model iteration, we decided to use the single landcover data source, NLCD (specifically, the 2016 data set) [36], for all states. This data source has a history of updating the data set unlike the data sources previously used for Colorado, New Mexico, and Texas. The Southwest Regional Gap Land Cover Dataset for Colorado and New Mexico had not been updated since 2001 and The Ecological Mapping Systems of Texas had not been updated since 2013. Additionally, concerns from state experts related to the quality of the NLCD 2011 product for Colorado and New Mexico were alleviated with improvements in the NLCD 2016 data set and another modification described below.

Lesser prairie-chickens’ use of grassland and shrubland habitats while excluding woodlands is well documented [21,37]. A major concern with originally using NLCD 2011 for New Mexico and Texas was NLCD 2011 classification of areas encroached by mesquite, which is unutilized by lesser prairie-chickens, as shrubland, which is utilized. We used a recently produced mesquite and red cedar encroachment layer (Playa Lakes Joint Venture, http://pljv.org/for-habitat-partners/maps-and-data/data-downloads/) to remove encroached areas from known/suspected used.

Percent Conservation Reserve Program lands within 5000 m radius, chosen for the same reason as known/suspected used radius, was also used as a predictor as these lands often contain suitable vegetation and were important in previous lek models [38]. These are lands enrolled in a program to remove environmentally sensitive lands from agricultural production and convert them to vegetative cover.

First iteration anthropogenic predictors based on 2011 information included roads, transmission lines, wells, and Federal Aviation Administration (FAA)-identified structures. Lesser prairie chicken avoidance of these types of features have been well documented [e.g., 14, 16, 19, 30]. In the second iteration, we used the latest updated versions of these data sets. However, the updated versions included not only new features constructed on the landscape but also removal of inaccurate features identified through quality checks (see Table 1 for specific dates and more information). We also used an updated version of the landscape condition index (2012 version used in first iteration), which is a human modification index based on transportation corridors, urban and industrial development, and managed/modified land cover data [31]. This predictor highlights features avoided by lesser prairie-chickens. Hak and Comer [22] created their updated predictor using different inputs and a different output scale than their 2012 version, making it impossible for us to directly assess changes between the two time periods.

Mean annual enhanced vegetation index (EVI; a spectral vegetation index that calculates photosynthetically active vegetation), incorporated in iteration one as a surrogate for resource availability [24], is no longer included as part of the NASA terra moderate resolution imaging spectroradiometer (MODIS) phenology product (MCD12Q2 v006). For the second iteration, we produced EVI with the 250 m resolution MODIS vegetation indices (MOD13Q1) version 6 product [39] within the Google Earth Engine platform [40]. We calculated annual mean EVI for each year within 2008–2017 (the most recent 10-year period available) and then averaged all years to create a decadal average EVI. As with landscape condition index, the values were not comparable between iterations as we were unable to directly replicate the NASA MCD12Q2 v006 algorithm.

### Maxent model iteration

We updated some components of model development from that previously used by Jarnevich et al. [24]. We used the newest version of the Maxent software for modeling species distributions, version 3.4.1 [41], within the Software for Assisted Habitat Modeling (SAHM; version 2.2.1) [42]. Maxent is a machine learning method that uses the principle of maximum entropy to identify relationships between available environment (as assessed via background points) and observed locations to predict habitat suitability at unknown locations [41,43,44]. Maxent is a commonly used method to create habitat suitability models when absence data are lacking. We input the updated predictors described above (Table 1). For presence data, there were 1,257 leks observed from 2015 to 2019 [45] by the different state agencies (see S1 Supporting Information for survey details), resulting in 1,012 pixels at 210 m resolution with at least one lek observation during the time period (compared to first iteration 2002–2012 n = 1,402 pixels at 210 m resolution). Thus, lek presence locations used included 12 in Colorado, 443 in New Mexico, 207 in Kansas, 284 in Oklahoma, and 66 in Texas. We generated 10,000 background points within the 16.1 km buffered estimated occupied range within which the lek surveys occurred using the *MDSBuilder* module within SAHM and a sampling bias layer, using a bias correction method sensu [46] rather than trying to directly model bias (e.g., [47]). The bias layer reflected the percentage of the EOR+10 area surveyed for leks by state with 30% for Colorado, 56% for New Mexico, 9% for Kansas, 80% for Oklahoma, and 34% for Texas as estimated by the Lesser Prairie-Chicken Interstate Working Group (including all non-USGS authors). A bias layer is needed to mimic bias existing in the presence data as these were not statistically collected and without a bias layer the model results may reflect sampling effort rather than habitat suitability [46,48]. Using this bias layer with the study area created 923 background points in Colorado, 2,928 in New Mexico, 790 in Kansas, 2,956 in Oklahoma, and 2,403 in Texas. Within SAHM we examined correlations among predictors for the updated source data, and all correlation coefficients (Pearson, Spearman, and Kendall) remained < +/-0.7, which is required to avoid issues from collinearity [49]. We used 10-fold cross-validation to evaluate model performance within SAHM, with the final model presented using all data points rather than averaging the 10 cross-validation models. We ran Maxent with default SAHM settings and examined response curves and the difference between training and average cross-validation area under the curve (AUC) as indicators of overfitting, setting a difference of 0.05 as an *a priori* cutoff for requiring changes. We also examined AUC to evaluate model performance.

As with the first iteration, we developed two sets of models. We ran models that both included and excluded state as a predictor to account for potential differences between states in their management strategies, treatment of Conservation Reserve Program (CRP) lands, and monitoring activities.

We used predictor importance from Maxent output, measured by both permutation importance (relativized measured change in AUC) when predictor values are randomly permutated between presence and background while other predictors are held constant) and percent contribution (model-dependent measure based on additive training gain at each iteration during model fitting). We also provide response curves produced by SAHM that are based on values for each predictor when other predictors are held constant.

We examined continuous relative habitat suitability index maps from Maxent, where the term ‘relative’ indicates that the results are a relative index of habitat suitability rather than a probability of occurrence due to the model reliance on presence data rather than presence-absence data. In addition to continuous maps, many applications of species distribution models use a binary map that discretizes the predictions [e.g., 50]. We provided discretized versions of map products based on three different threshold rules following Jarnevich et al. [24] to allow practitioners to select context-specific costs of false positives (i.e., missing habitat that is actually suitable in the final habitat suitability map). These thresholds include the minimum training presence (MTP; value is the minimum prediction for any training presence location), fifth percentile (orders prediction values for all training presence locations and selects value that would classify the bottom 5% as unsuitable), and tenth percentile (orders prediction values and misclassifies the bottom 10%). We created maps based on these three thresholds and calculated the amount of predicted suitable lek habitat within the study area, the estimated occupied range, and ecoregions in R v3.6.0 [51] using the raster package [52] and the tidyverse package [53]. All predictors and model raster outputs are available through the Science Base data repository [54].

### Habitat suitability comparison

We intended to apply the Jarnevich et al. [24] Maxent models to the updated predictors. However, because of barriers to direct comparison described above, we used an alternative to directly examine land cover changes that could impact lek habitat suitability. We compared the amount of the landscape in the three known/suspected used classes in the NLCD 2016 product version of land cover for 2011 to land cover for 2016. This method allowed a direct comparison of landscape land cover changes as these two layers are from the same source and are produced using the same methodology. We also compared the predictions from Jarnevich et al. [24], which are being used by decision makers, to our second iteration models within the 2016 study extent to see how predictions changed between the two model iterations, realizing that differences could arise due to methodological differences, quality of the predictors, and changes on the landscape.

## Results

### Maxent model

Average EVI followed by percent known/suspected used land cover within 5,000 m contributed the most to predicting habitat suitability in the second iteration models both with and without state (S1 Fig). Excluding state, the top four predictors were the same for the second iteration models as measured by Maxent computation of permutation importance. When state was included, it was the third-most important predictor in the model, with Kansas having the highest suitability.

Response curves illustrate general trends in the relationship between each predictor and lek suitability. The responses were similar in models both with and without state, with suitability values being dampened with models including state (S1 Fig). There was a general trend of increasing suitability with increasing percent known/suspected used, percent CRP and landscape condition index. Suitability quickly dropped off as well density increased whereas suitability was predicted to be higher across a broader range of distance from transmission lines, roads, highways, and FAA structures. Individually, these anthropogenic features had a low contribution to the model, except for well density as measured by Maxent permutation importance. Viewed together as anthropogenic impacts, they contributed 14.0% and 19.3% to runs with state and without state, respectively.

**Fig 2:**
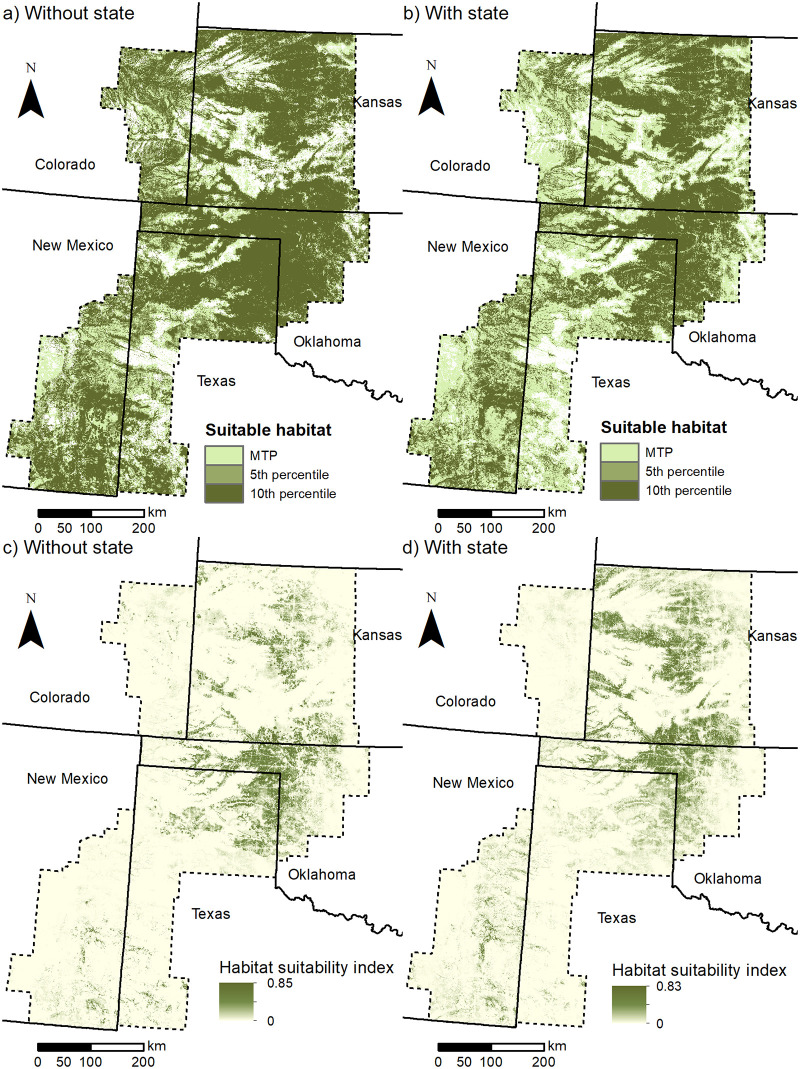
Lek suitability model predictions by threshold and predictor set. Lesser prairie-chicken lek suitability model predictions without state boundary as a predictor (a, c) and including state boundary as a predictor (b, d) displayed as (a-b) the three different suitability thresholds used to classify the models into suitable and unsuitable habitat including minimum training presence (MTP), 5^th^ percentile, and 10^th^ percentile and (c-d) the continuous habitat suitability index, displayed using a minimum maximum stretch method. [Projected Coordinate System: Albers Conic Equal Area].

**Fig 3:**
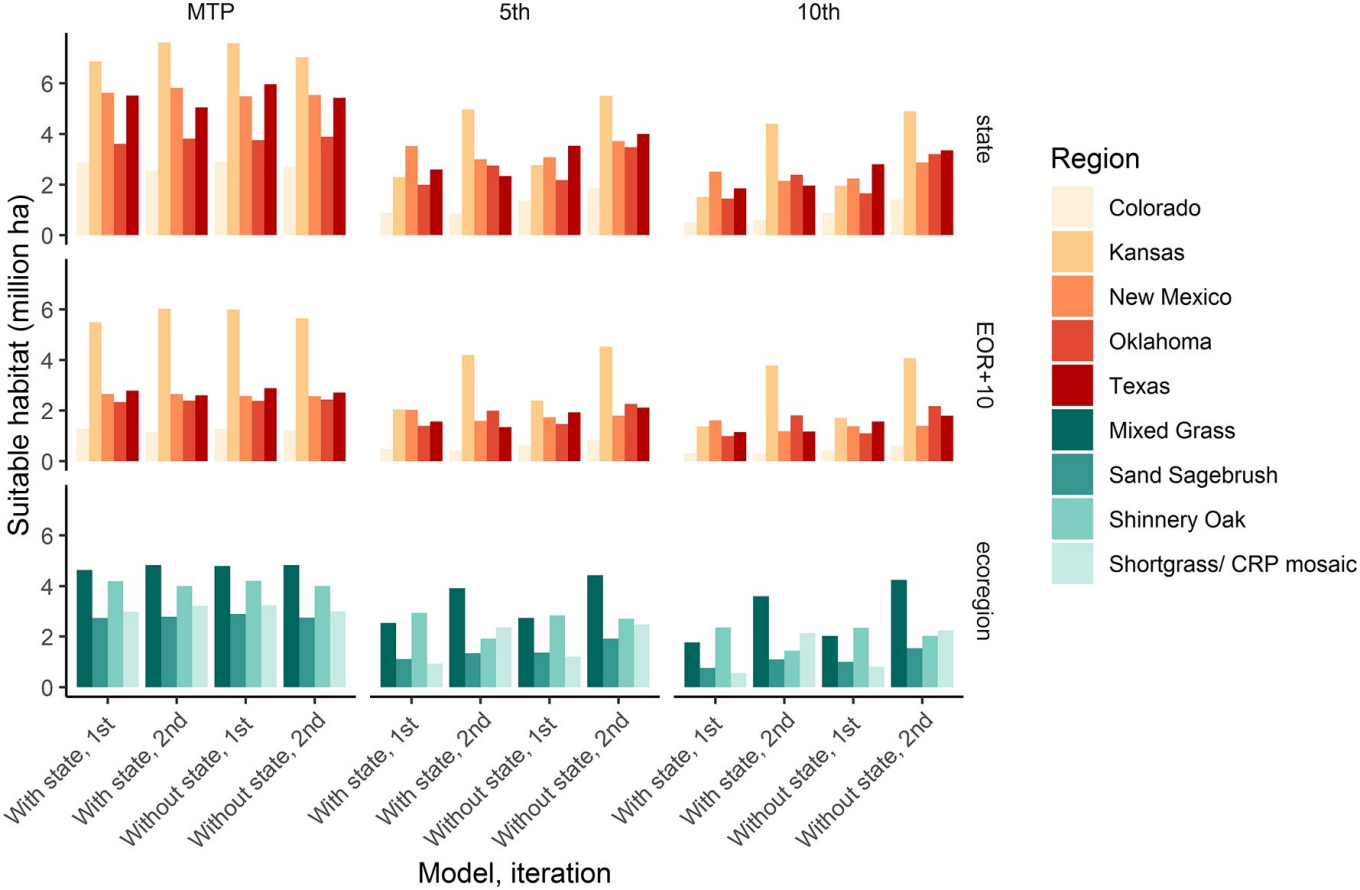
Comparison of area predicted suitable by threshold, predictor set, state, study extent, and ecoregion. Lesser prairie-chicken lek suitable habitat predictions for different thresholds (columns) including minimum training presence (MTP), 5^th^ percentile, and 10^th^ percentile and different region types (rows) including state, buffered estimated occupied range (EOR+10), and ecoregion. Bars are grouped by the model (with or without state included as a predictor) and iteration (first or second version).

Despite the similarities in the two second iteration models (with and without state), there were large differences in suitability predictions across the study area when state was included as a predictor (Figs 2 and 3 and S1 Table). The differences were most apparent for Texas, which had markedly less suitable habitat at higher thresholds when state was included (with state had 484,531 [MTP], 2,290,091 [5 percentile], and 1,926,204 [10 percentile] ha less). The models without state included had more suitable habitat across all thresholds for all states except Kansas and New Mexico for the MTP threshold. For models without state, predicted suitable habitat in the buffered estimated occupied range was generally higher than the first iteration estimates [24], particularly for the 5 and 10 percentile thresholds. Models with state had more variability in pattern of predicted suitable area within the estimated occupied range between iterations. There was a similar relationship in area predicted suitable in the entire study area (Table 2). Among ecoregions, Mixed Grass had the highest suitable area across thresholds and models, followed by Shortgrass/CRP mosaic and Shinnery Oak (mixed as second or third), with Sand Sagebrush having the lowest suitable area (S2 Table and Fig 3). Shinnery Oak decreased in suitable area from the first iteration to the second, while the others all increased except MTP for the model without state for Sand Sagebrush and Shortgrass/CRP mosaic.

**Table 2:** Metrics comparing area of suitable habitat modeled with and without state as a predictor.

Model	AUC		Threshold method	Threshold value	Study area suitable (Suitability change) [ha]	EOR+10 area suitable (Suitability change) [ha]
	Test	Train				
**With State**	0.81	0.81	MTP	0.0002	28,693,101 (+372,447)	14,835,272 (+285,024)
			5 per.	0.0084	16,095,406 (+2,605,190)	9,537,136 (+2,014,090)
			10 per.	0.0167	13,330,142 (+3,691,576)	8,265,642 (+2,824,333)
**Without State**	0.71	0.72	MTP	0. 00004	28,521,583 (-1,088,661)	14,572,311 (-543,228)
			5 per.	0.0004	21,616,404 (+5,628,527)	11,516,918 (+3,366,294)
			10 per.	0.0011	18,335,960 (+6,223,604)	10,046,157 (+3,867,178)

Suitability change, in parentheses, depicts the net change in suitable area between the first [24] and second iteration, for study area and estimated occupied range buffered by 10 mi (EOR+10) area [8]. AUC = area under the curve; MTP = minimum training presence, 5 per. = 5 percentile, and 10 per. = 10 percentile.

### Habitat suitability comparison

There were several differences between the first iteration predictors and the second iteration predictors. Overall, there was a change in the amount of the landscape in the individual land cover types classified as known/suspected used, but the total amount in these three land cover types only decreased slightly (Table 3; <1% decrease). This small net loss between NLCD 2011 and NLCD 2016, however, does not account for the addition of the woody encroachment, which overlaid with almost 1.5 million hectares of known/suspected used cover classes primarily Mixed Grass and Shinnery Oak ecosystems (Table 3b and 3c). The small net loss also does not reflect the greater number of locations that switched cover classes, where some locations became classes classified as unused while others became classes classified as known/suspected used (Table 3b). All states except Colorado had a net loss of more than 350,000 ha of known/suspected used land cover for a total loss of 1,718,473 hectares across the study area, which is largely accounted for by woody encroachment. Overall, Kansas had the most stable area of known/suspected used land cover in the study region between 2011 and 2016. Within the buffered estimated occupied range, all ecoregions experienced a net loss in known/suspected used land cover based on changes in NLCD (Table 3c), with the greatest decrease in the Mixed Grass and Shinnery Oak ecoregions.

**Table 3:** Changes in area of land cover categories by study area, state, and ecoregion and state between first and second iteration modeling for lesser prairie chickens.

a)						
**NLCD class**	**Study Area**			**EOR+10**		
	**NLCD 2011 area (ha)**	**NLCD 2016 area (ha)**	**Percent change from 2011**	**NLCD 2011 area (ha)**	**NLCD 2016 area (ha)**	**Percent change from 2011**
Shrubland (Dwarf Scrub and Shrub/Scrub)	8,303,583	8,536,449	0.71	3,790,531	3,889,898	0.30
Grassland/ Herbaceous	11,921,851	11,385,317	-1.63	6,080,672	5,837,459	-0.74
Pasture/Hay	215,859	228,281	0.04	151,728	161,776	0.03
Known/ suspected used	20,441,292	20,150,047	-0.89	10,022,931	9,889,133	-0.41
EOR+10 area is the estimated occupied range buffered by 10 miles [8]; NLCD = National Land Cover Dataset.						

b)					
**Study area by state**	**2016 Known/suspected used encroached by woody species (ha)**	**Switch from known/suspected used (ha)**	**Stable (ha)**	**Switch to known/suspected used (ha)**	**Net change in known/suspected used (ha)**
Kansas	317,285	399,614	10,006,702	5,714	-393,900
New Mexico	414,204	449,859	5,691,089	2,966	-446,893
Oklahoma	350,679	389,290	3,858,765	3,773	-385,517
Texas	345,079	459,904	7,951,061	7,633	-452,271
Colorado	6	40,867	3,597,996	974	-39,893
Total	1,427,253	1,739,533	31,105,613	21,060	-1,718,473
Change from NLCD 2016 known/suspected used based on woody encroachment (column 2) and changes between known/suspected used classification (columns 3–6) for each state within the study area. NLCD = National Land Cover Dataset.					

c)					
**EOR+10 by Ecoregion**	**2016 Known/suspected used encroached by woody species (ha)**	**Switch from known/suspected used (ha)**	**Stable (ha)**	**Switch to known/suspected used (ha)**	**Net change in known/suspected used (ha)**
Mixed Grass	337,080	373,793	4,739,073	4,776	-369,017
Sand Sagebrush	8,669	37,513	3,212,979	1,425	-36,088
Shinnery Oak	229,564	279,932	4,181,581	3,805	-276,127
Shortgrass/CRP mosaic	63,013	92,496	3,404,692	1,601	-90,895
Total	337,080	783,734	15,538,325	11,607	-772,127
Change from NLCD 2016 known/suspected used based on woody encroachment (column 2) and changes between known/suspected used classification (columns 3–6) for each ecoregion within the EOR+10 area (estimated occupied range buffered by 10 miles [8]). NLCD = National Land Cover Dataset.					

It is difficult to directly attribute changes in habitat suitability between the model iterations to changes on the landscape, but we can still assess changes in predicted habitat suitability. The second iteration models predicted a greater percentage of the estimated occupied range as suitable than the first iteration models both overall (Table 2) and by state (Fig 3 and S2A Table). Given the small difference in change noted between the two NLCD time periods (Table 3) relative to the large first and second iteration known/suspected used difference for Colorado, New Mexico, and Texas (S3B Fig), changes in predicted habitat suitability for these states may reflect the difference in land cover products more than actual changes on the landscape. For ecoregions, there were decreases for the Shinnery Oak Ecoregion for all models and thresholds (Fig 3 and S2B Table; roughly similar to loss in predicted suitable habitat), while there were also decreases for the models without state with the MTP threshold for the Sand Sagebrush and the Shortgrass/CRP mosaic Ecoregions.

## Discussion

Due to the issues previously discussed it was not possible to directly assess the changes in lek habitat suitability that occurred between the generation of the first and second iteration models. The inability to assess direct changes due to changes in data availability and cleaning of errors in original predictor data contributed to these issues. These issues suggest that it may not be simple for researchers to provide models to managers that could simply be applied to temporally updated input predictors unless a model is originally developed with this type of iteration in mind. The goal of the first iteration was to produce the best model with the best available data. Through that effort, alternative predictor data sets were explored, and the best data were chosen for different parts of the range. These data sets were then modified to make them comparable so that a range-wide predictor could be developed. These decisions resulted in complicated predictor production, such as three different land cover data sources that needed to be reconciled into a single predictor, with each data set having differing approaches to temporal updates.

We set out to create a second iteration, assuming we would be able to obtain temporally updated predictors, such as the time sequenced NLCD data set, and apply the first iteration model to the updated predictors as is often done for climate change assessments. Our assumption was incorrect due to lack of consistent temporal updates of predictors, except for percent CRP. Even the EVI predictor produced by a NASA program was discontinued as a product and we were unable to replicate its production. Thus, we fit a new model with recent lek survey data and associated updated predictor variables. We attempted to revise predictors in a way that would allow for direct comparison in future iterations. We reduced the land cover data to a single source that has been regularly updated for more than two decades (NLCD). The EVI predictor is now calculated in google earth engine base on raw satellite imagery, so we can easily produce a comparable version for a different time. Other changes may be harder to track, but less important in iterating, such as changes from data cleaning of anthropogenic features that were indistinguishable from on-the-ground changes (see red colors in S3 Fig), as all data sources likely have unknown errors. However, there is a trade-off between making models directly comparable and including the best data available at the time. For example, the addition of woody encroachment that was unavailable for the first iteration proved highly important in understanding what changes may have occurred in land cover.

Despite these challenges, we were able to directly evaluate changes in land cover, which was an important predictor of habitat suitability, using the NLCD 2016 product for 2011 and 2016. While there was only a small net change in known/suspected used land cover classes, the decrease in the grassland/herbaceous category for our study area and for the buffered estimated occupied range could be important because lesser prairie-chicken rely on a high ratio of grassland to agricultural land for resilience to stochastic events such as drought [55]. Excluding woody encroachment from the percent known/used predictor due to the avoidance of grasslands with trees by lesser prairie-chickens [21] and other similar prairie grouse species resulted in further reduced known/suspected used land cover in the NLCD 2016 derived predictor. Even these spatial layers likely underestimate the influence of trees given the inability to capture small/low density stands of trees using this scale of imagery. Despite issues of direct comparability, we were able to provide information on changes in predictors including land cover and to develop a second iteration model to provide updated lek habitat suitability.

The response curves illustrating the relationships found between predictors and lek habitat suitability in the Jarnevich et al. [24] models followed similar patterns to those found in other efforts [38,56,57], and these patterns remained consistent in this second iteration of modeling. These relationships point to avoidance of anthropogenic features, which match studies of habitat selection and space use [e.g., 58, 59]. Conservation reserve program lands were also important in both lek suitability model iterations, matching others’ findings of their importance as habitat for lesser prairie-chickens, at least in the northern part of their range [60–63].

The first iteration model included several more lek locations as training data (first iteration n = 1,402; second iteration n = 1,012) with the greatest difference in Colorado and Texas lek counts. We chose to restrict the period for lek occurrences to 2015 to 2019 to avoid issues in pseudo-replication between years and match the rough time frame of predictors. Additionally, the Southern High Plains experienced the worst drought on record in 2011, resulting in reduced lesser prairie-chicken abundance in the region that had not recovered by 2015 [64]. This drought could have led to the reduced lek count range-wide [65], although abundances have generally increased from the drought condition population [66]. The dampened suitability in Texas with state as a predictor may also have been related to the response of the species’ population to drought. The Sand Sagebrush Ecoregion had the lowest amount of suitable habitat among the ecoregions, matching the finding of a high likelihood of extirpation risk for the region [66], though models predicted an increase in suitability between the iterations. For this ecoregion, however, the land cover data source changed between the first and second iteration, so increases do not necessarily reflect actual changes on the landscape, particularly since Sand Sagebrush experienced a net loss in known/suspected used land cover between NLCD 2011 and NLCD 2016. The updated model provides an updated prediction of potential habitat using recent lek survey data (2015–2019) and predictors from the same time frame. The predictor sources were chosen with iterative modeling in mind to facilitate future updates that would allow direct comparisons of changes.

The second iteration models were based on the same suite of predictors chosen for the first iteration by a group including managers after testing various sets of predictors, and we did not revisit alternative predictors. Both iterations were based on habitat suitability for a single habitat use that is well-observed range-wide, lekking, to infer general habitat requirements for the species. Lek occurrence has been used as a surrogate for other life stages that may be more limiting to the population given lack of consistent range-wide data for other activities such as nesting [38]. These models also do not capture spatial-temporal dynamics that have been investigated at smaller scales within the range [55,67].

We evaluated our model following the assessment rubric provided by Sofaer et al. [1] to communicate model quality to end users by rating categories related to species data, predictors, modeling process, and model products as “interpret with caution”, “acceptable”, or “ideal” for a particular model. This second iteration model had a ranking of either acceptable or ideal in all categories of the model evaluation rubric [S2 Table; 1]. This table should provide end users with information to determine if the model is appropriate for their intended use.

## Conclusions

Predicted lek habitat suitability has changed across the study area in recent years, though directly assessing changes proved impossible. We provide lessons learned for other iterative modeling projects that rely on predictors that are not consistently updated, noting a need to balance best-available data with temporal consistency. Products such as NLCD are useful in that they provide consistent methodology to develop products for different time periods. Accounting for woody encroachment produced an overall decrease in land cover types known/suspected used for lekking, when compared to the first iteration model. There was an overall increase in important land cover types when ignoring woody encroachment. Likely the 2011 NLCD overestimated known/suspected used land cover classes as some of these areas were likely encroached by woody vegetation. The second iteration models generally predicted more suitable habitat across both the study area and the buffered estimated occupied range than the first iteration model predictions. Regardless of the underlying reason for changes in predicted suitable habitat, these models provide updated information about lek habitat suitability across the range. These predictions, based on more recent predictor data, can be used to update decision support tools such as the Southern Great Plains Crucial Habitat Assessment Tool where the predications could be used to help determine siting of development [such as 38] or where corridors may be needed between populations to facilitate connectivity between ecoregions [66].

While species distribution models applied to future climate predictions or land use scenarios are common, near-term iteration of models are not. Periodically iterating models with current information about the landscape and other predictors allows the models and the tools relying on them to remain current and useful for decision makers, despite issues in iteration due to inconsistent updating of important predictors. Iterations also allow predictions to be improved by incorporating newly available information and data sets such as the woody encroachment information used in this example.

## Supporting information

S1 Fig Predictor response curves. Plots are ordered by permutation importance, showing relative lesser prairie-chicken lek habitat suitability (y-axis) across the range of values in the occurrence data for each predictor (x-axis). The numbers in the top left of each graph represent the permutation importance (percent contribution) from Maxent for that predictor for the model run with state and without state, respectively. The red lines along the x-axis represent presence points with those values. (EVI = enhanced vegetation index; FAA = federal aviation administration).
(JPG)

S2 Fig Changes in habitat suitability predictions from the first to second modeling iteration. The top row of figures (a-c) compares first and second iteration predictions for models including state as a predictor, while the bottom row compares predictions from models that do not include state as predictor (d-f). Comparisons were made for the three thresholds, minimum training presence (MTP) threshold (a, d), fifth percentile (b, e), and tenth percentile (c, f). Note that the change in land cover data source underlying the percent known/s/suspected used predictor for Colorado, New Mexico, and Texas between the two iterations of the model may account for some changes in suitability (Table 1). [Projected Coordinate System: Albers Conic Equal Area].
(JPG)

S3 Fig Comparison of predictor versions between first and second modeling iterations. Sources for all data can be found in Table 1. S3A Fig. Comparison of features used to create anthropogenic predictors in first and second modeling iterations. Purple denotes presence in both iteration predictors; red denotes presence in the first iteration predictor but not the second (likely the result of data cleaning); and blue indicates a new feature in the second iteration predictor. Updated spatial data includes a) transmission lines, b) highways, c) secondary roads, d) vertical structures over 50m, and e) active wells (see Table 1 for sources). [Projected Coordinate System: Albers Conic Equal Area]. S3B Fig. Difference between used and updated raster values in predictors used to model lesser prairie chicken habitat suitability. Values represent the original predictor subtracted from the updated predictor, so that red colors represent an increase and the blue colors represent a decrease in value from the original predictor. Updated data includes a) percent conservation reserve program (CRP) land area within a 5000 m neighborhood, b) average of the average Enhanced Vegetation Index (EVI) for 2000–2009 (recreated original predictor following updated protocol) and 2008–2017 (updated predictor), and c) the percent area known/suspected used by lesser prairie-chickens in a 5000-m neighborhood (note land cover data sources for Colorado, New Mexico, and Texas changed between the two iterations and the change may account for many differences in those states). See Table 1 for all sources. [Projected Coordinate System: Albers Conic Equal Area].
(PDF)

S1 Table Comparison of area classified as suitable by threshold, predictor set, state, study extent, and ecoregion. (PDF)

S2 Table Model assessment rubric. From Sofaer et al. [1] for first iteration model [24] and the second iteration model.
(PDF)

S1 File Prairie chicken lek survey methodology by state. (PDF)
