# Supplementary figures and images for: Challenges in updating habitat suitability models: An example with the lesser prairie-chicken
Source: PLoS One. 2021 Sep 20;16(9):e0256633. doi: 10.1371/journal.pone.0256633 (PMC8452035; doi:10.1371/journal.pone.0256633)

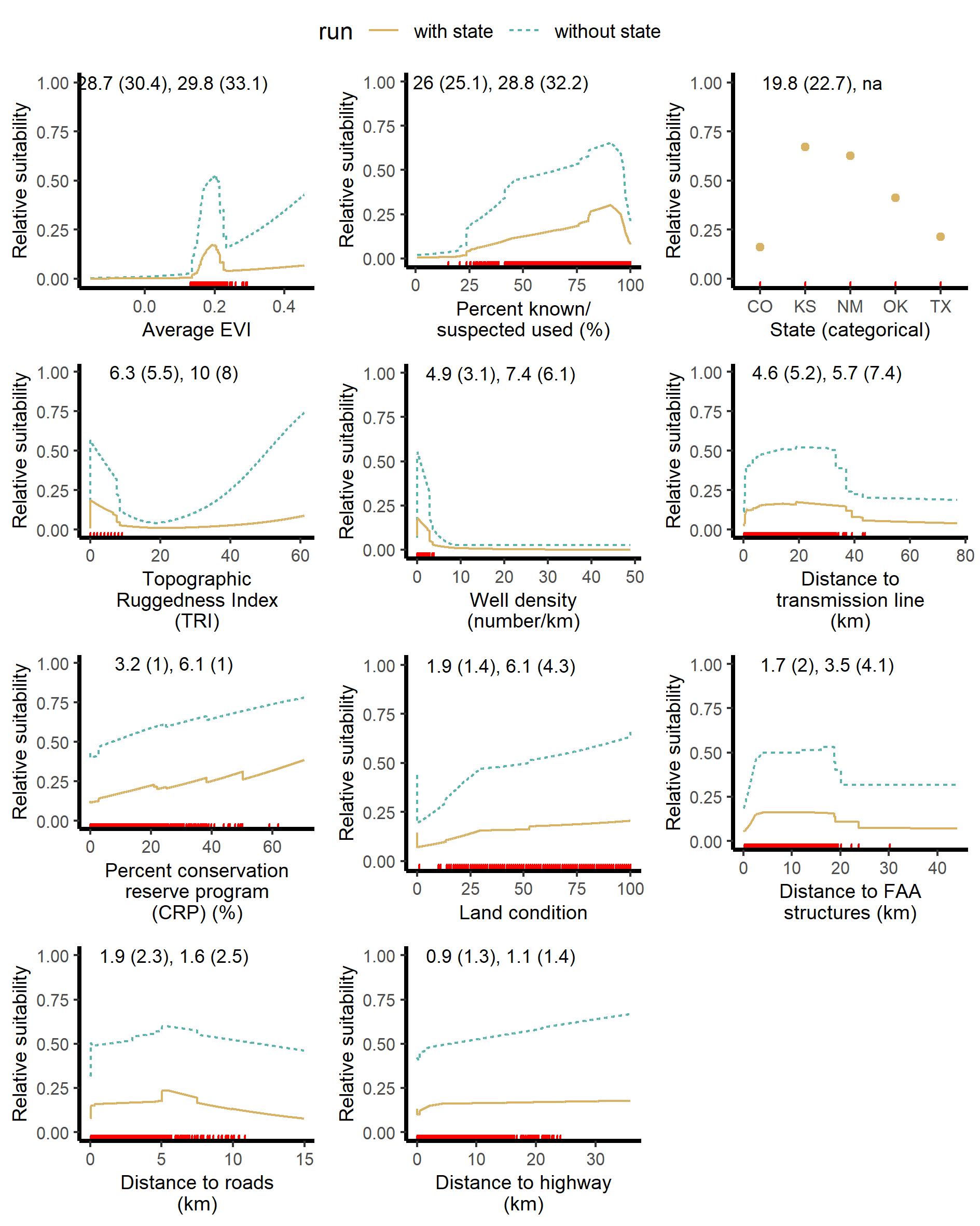

Supplement: S1 Fig — Plots are ordered by permutation importance, showing relative lesser prairie-chicken lek habitat suitability (y-axis) across the range of values in the occurrence data for each predictor (x-axis). The numbers in the top left of each graph represent the permutation importance (percent contribution) from Maxent for that predictor for the model run with state and without state, respectively. The red lines along the x-axis represent presence points with those values. (EVI = enhanced vegetation index; FAA = federal aviation administration). (JPG) [file pone.0256633.s001.jpg]

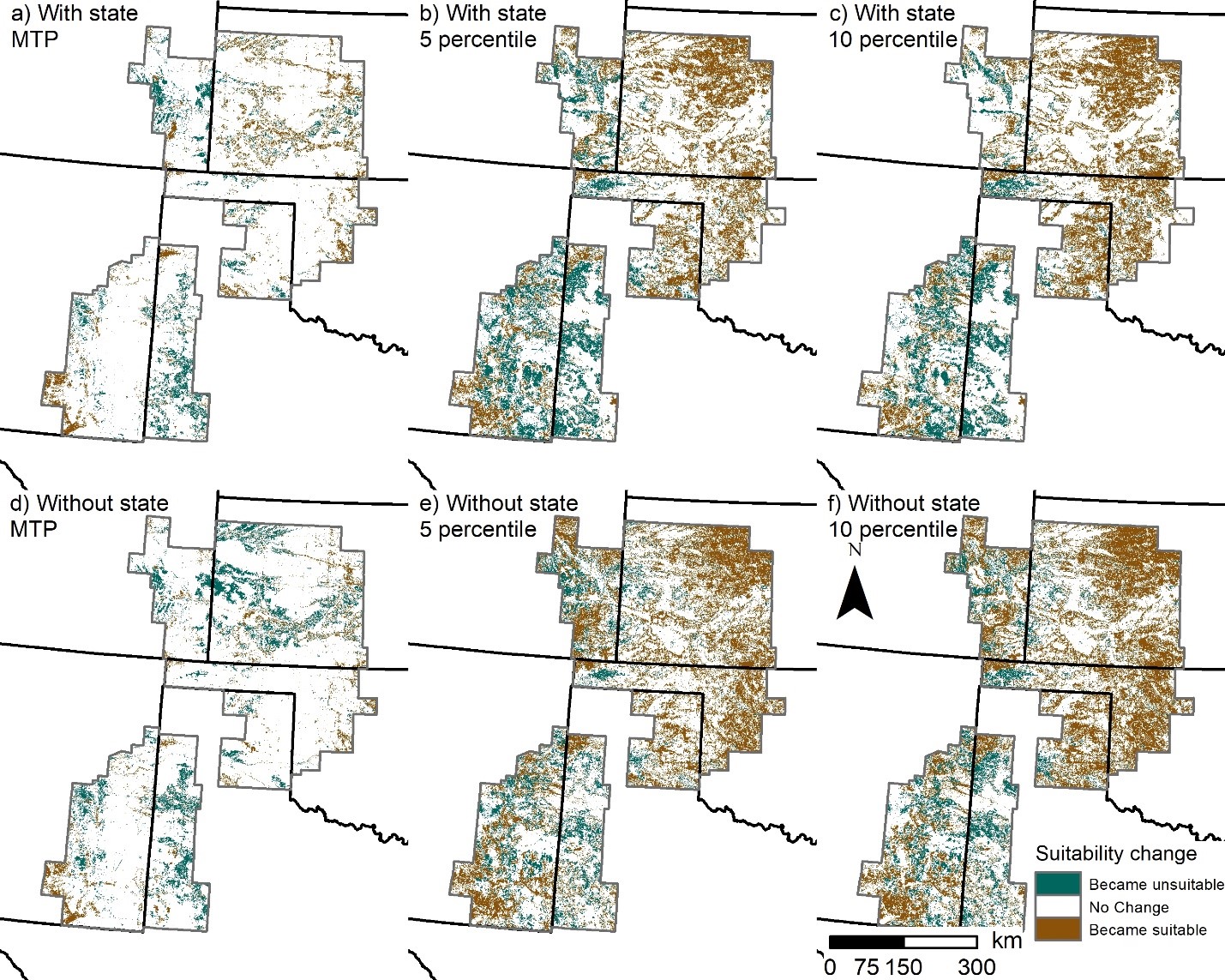

Supplement: S2 Fig — The top row of figures (a-c) compares first and second iteration predictions for models including state as a predictor, while the bottom row compares predictions from models that do not include state as predictor (d-f). Comparisons were made for the three thresholds, minimum training presence (MTP) threshold (a, d), fifth percentile (b, e), and tenth percentile (c, f). Note that the change in land cover data source underlying the percent known/s/suspected used predictor for Colorado, New Mexico, and Texas between the two iterations of the model may account for some changes in suitability (Table 1). [Projected Coordinate System: Albers Conic Equal Area]. (JPG) [file pone.0256633.s002.jpg]

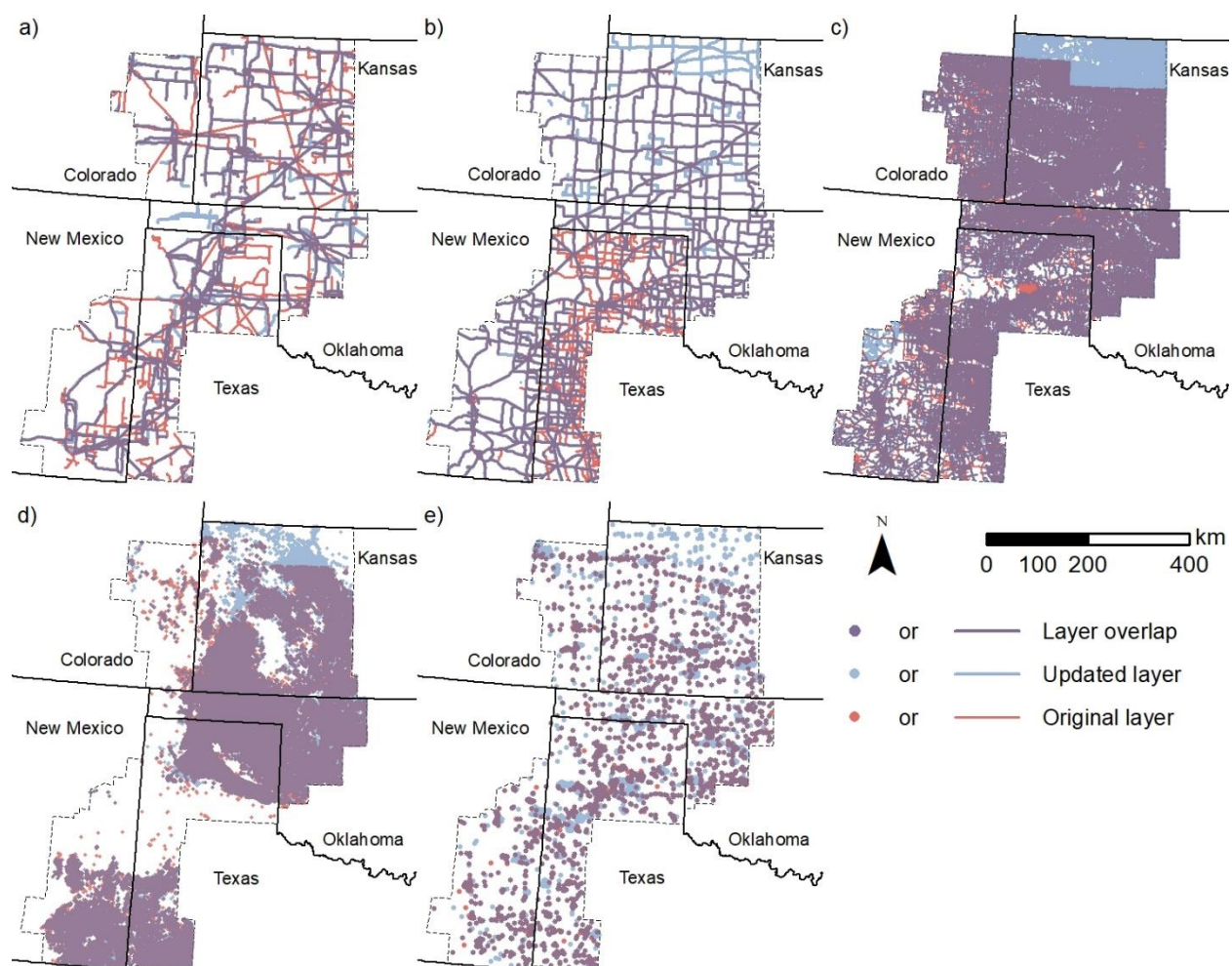

**S3A Fig.**

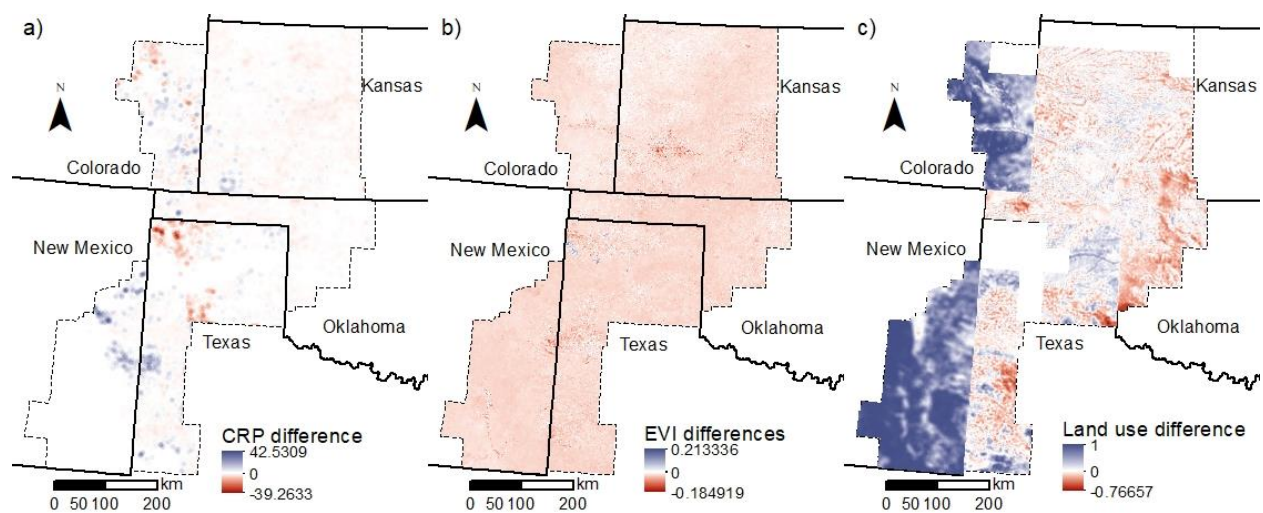

**S3B Fig.**

Supplement: S3 Fig — Sources for all data can be found in Table 1. S3A Fig. Comparison of features used to create anthropogenic predictors in first and second modeling iterations. Purple denotes presence in both iteration predictors; red denotes presence in the first iteration predictor but not the second (likely the result of data cleaning); and blue indicates a new feature in the second iteration predictor. Updated spatial data includes a) transmission lines, b) highways, c) secondary roads, d) vertical structures over 50m, and e) active wells (see Table 1 for sources). [Projected Coordinate System: Albers Conic Equal Area]. S3B Fig. Difference between used and updated raster values in predictors used to model lesser prairie chicken habitat suitability. Values represent the original predictor subtracted from the updated predictor, so that red colors represent an increase and the blue colors represent a decrease in value from the original predictor. Updated data includes a) percent conservation reserve program (CRP) land area within a 5000 m neighborhood, b) average of the average Enhanced Vegetation Index (EVI) for 2000–2009 (recreated original predictor following updated protocol) and 2008–2017 (updated predictor), and c) the percent area known/suspected used by lesser prairie-chickens in a 5000-m neighborhood (note land cover data sources for Colorado, New Mexico, and Texas changed between the two iterations and the change may account for many differences in those states). See Table 1 for all sources. [Projected Coordinate System: Albers Conic Equal Area]. (PDF) [file pone.0256633.s003.pdf]
