# Supplementary material for: Challenges in updating habitat suitability models: An example with the lesser prairie-chicken
Source: PLoS One. 2021 Sep 20;16(9):e0256633. doi: 10.1371/journal.pone.0256633 (PMC8452035; doi:10.1371/journal.pone.0256633)
Supplement: S1 Table — (PDF) [file pone.0256633.s004.pdf]

a)

Model	State	Area suitable (Area change from 2016) [ha]			EOR+10 suitable (Area change from 2016) [ha]		
		MTP	5 per.	10 per.	MTP	5 per.	10 per.
**Without state**	CO	2,551,899 (-309,093)	847,298 (-23,046)	594,874 (101,179)	1163671 (-123026)	418491 (-77387)	317189 (12948)
	KS	7,611,378 (738,711)	4,966,105 (2,662,841)	4,406,772 (2,897,670)	6022120 (537231)	4188098 (2142427)	3774157 (2400173)
	NM	5,820,697 (193,705)	2,991,360 (-532,883)	2,147,044 (-360,967)	2648068 (-6201)	1591137 (-436824)	1188319 (-430592)
	OK	3,810,990 (208,412)	2,757,101 (757,589)	2,389,113 (942,580)	2395525 (55808)	1989946 (601453)	1811584 (816900)
	TX	5,051,068 (-459,289)	2,339,897 (-259,313)	1,959,628 (111,115)	2605891 (-178795)	1349464 (-215579)	1174392 (24903)
**With state**	CO	2,706,130 (-191,315)	1,850,070 (505,518)	1,413,886 (541,773)	1211030 (-68968)	817102 (188289)	612183 (191271)
	KS	7,033,050 (-542,933)	5,497,850 (2,735,876)	4,885,094 (2,936,716)	5647869 (-340845)	4526605 (2134471)	4067815 (2357837)
	NM	5,544,208 (54,953)	3,717,745 (632,871)	2,877,221 (633,135)	2566633 (-13155)	1793719 (62600)	1397573 (14407)
	OK	3,884,698 (127,493)	3,473,175 (1,294,000)	3,205,073 (1,554,304)	2428891 (47650)	2267207 (802628)	2170748 (1073756)
	TX	5,427,268 (-536,860)	3,995,897 (460,263)	3,354,581 (557,675)	2717892 (-167906)	2112289 (178310)	1797838 (229907)
**Difference**	CO	175,589	1,119,572	915,618	11,271	90,388	66,892
	KS	653,161	589,202	541,672	104,840	122,175	119,657
	NM	277,799	726,671	730,957	19,484	84,049	89,448
	OK	99,322	795,462	891,367	9,712	62,871	81,475
	TX	484,531	2,290,091	1,926,204	28,279	173,024	141,407

Area changed, in parentheses, reports the difference in suitable area within specified areas between the first [24] and second model iterations. MTP = minimum training presence, 5 per. = 5 percentile, and 10 per. = 10 percentile.

b)

		EOR+10 suitable (change) [ha]		
**Model**	**EOR+10 by ecoregion**	**MTP**	**5 per.**	**10 per.**
**Without state**	Mixed Grass	4,827,561 (42,257)	4,426,661 (1,684,408)	4,241,026 (2,212,342)
	Sand Sagebrush	2,752,735 (-137,967)	1,912,652 (544,295)	1,536,334 (533,769)
	Shinnery Oak	3,998,256 (-199,601)	2,696,314 (-140,039)	2,021,928 (-322,732)
	Shortgrass/ CRP mosaic	2,993,759 (-247,917)	2,481,291 (1,277,630)	2,246,869 (1,443,799)
**With state**	Mixed Grass	4,827,892 (196,263)	3,911,308 (1,373,415)	3,591,522 (1,824,051)
	Sand Sagebrush	2,789,493 (50,795)	1,346,488 (236,888)	1,102,853 (349,771)
	Shinnery Oak	3,999,182 (-197,418)	1,918,085 (-1,017,873)	1,436,584 (-922,532)
	Shortgrass/ CRP mosaic	3,218,705 (235,384)	2,361,255 (1,421,660)	2,134,683 (1,573,043)

Area changed, in parentheses, reports the difference in suitable area within specified areas between the first [24] and second model iterations. MTP = minimum training presence, 5 per. = 5 percentile, and 10 per. = 10 percentile.
