# Supplementary material for: Challenges in updating habitat suitability models: An example with the lesser prairie-chicken
Source: PLoS One. 2021 Sep 20;16(9):e0256633. doi: 10.1371/journal.pone.0256633 (PMC8452035; doi:10.1371/journal.pone.0256633)
Supplement: S2 Table — From Sofaer et al. [1] for first iteration model [24] and the second iteration model. (PDF) [file pone.0256633.s005.pdf]

		Jarnevich et al. 2016	Current models
Species Data	Presence data quality	Acceptable: Location data evaluated for accuracy.	Acceptable: Location data evaluated for accuracy, collected with similar protocols.
	Absence/ background data	Acceptable: Background data selected within certain distance of presences; inconsistent biases by state.	Acceptable: Background data selected based on state biases in sampling of range; similar protocol used across states.
	Evaluation data	Acceptable: Cross-validation of training data.	Acceptable: Cross-validation of training data.
Environmental Predictors	Ecological and predictive relevance	Acceptable: Predictors chosen based on expert knowledge of species.	Acceptable: Same predictors as previous iteration but updated temporally.
	Spatial and temporal alignment	Acceptable: Predictors match sampling period as closely as possible. Used available resolution closest to that desired for mapped products.	Acceptable: Predictors match sampling period even more closely. Used available resolution closest to that desired for mapped products.
Modeling Process	Algorithm choice	Acceptable: Used maxent as had presence-only data.	Acceptable: Used maxent as had presence-only data.
	Sensitivity	Acceptable: Evaluated alternative background generation methods and predictor sets. Analyzed parameter settings based on *a priori* criteria.	Acceptable: Followed methodology from previous model and analyzed parameter settings separately based on *a priori* criteria.
	Statistical rigor	Acceptable: Examined collinearity issues and visually evaluated residual map for spatial patterns.	Acceptable: Examined collinearity issues and visually evaluated residual map for spatial patterns.
	Performance	Acceptable: Evaluated multiple evaluation metrics to ensure they met *a priori* criteria. Visually examined mapped products to evaluate ecological plausibility.	Acceptable: Evaluated multiple evaluation metrics to ensure they met *a priori* criteria. Visually examined mapped products to evaluate ecological plausibility.
	Model review	Acceptable: Review by species experts.	Acceptable: Review by species experts.
Model Products	Mapped products	Acceptable: Continuous map plus three different thresholds maps.	Acceptable: Continuous map plus three different thresholds maps.
	Interpretation support products	Ideal: Model attributes described. Mangers engaged in development of models and the format of delivery.	Ideal: Followed previous work.
	Reproducibility	Acceptable: Inputs, scripts, settings, and results available. Layers were not updateable to new time periods.	Ideal: Inputs, scripts, settings, and results available.
	Iterative	Interpret with caution: First iteration	Acceptable: Second iteration, with models easier to apply to future updated predictors.
