# Supplementary material for: Challenges in updating habitat suitability models: An example with the lesser prairie-chicken
Source: PLoS One. 2021 Sep 20;16(9):e0256633. doi: 10.1371/journal.pone.0256633 (PMC8452035; doi:10.1371/journal.pone.0256633)
Supplement: S1 File — (PDF) [file pone.0256633.s006.pdf]

S1 Supporting Information. Prairie chicken lek survey methodology by state.

Colorado

Two primary survey methods were used in 2016: known lesser prairie-chicken (LPC) lek surveys, and ad hoc surveys. Known leks included active leks (active at least once in last 5 years), inactive leks (active within 10 years but not in last 5 years), and historic leks (not active within the last 10 years). The goal for the known lek surveys was to survey as many active (active at least once in last 5 years), inactive (active within 10 years but not in last 5 years), and historic (not active within the last 10 years) leks as possible, under the following parameters:

- When possible, each lek was surveyed on at least two separate mornings.
- The lek survey period began as early as 30 minutes prior to sunrise and would continue for as long as two hours after sunrise.
- The surveyor would listen for at least 5 minutes from a distance no greater than 0.5 miles from the lek being surveyed.
- Surveys were conducted on mornings with low wind (<10 mph. based upon Beaufort scale) and no precipitation.
- If birds were detected, surveyors would approach the lek and count the numbers of males and females present. When appropriate/necessary the birds were flushed off the lek and counted.
- Surveys were conducted between March 15th and May 15th, with most surveys completed during the month of April.

The purpose of the ad hoc surveys was to locate new LPC leks. Ad hoc surveys were conducted by vehicle and on foot. In many cases surveys were carried out along county roads by stopping every ~0.5 miles through suitable chicken habitat areas and listening for a minimum of 5 minutes. Other surveys were held on specific ranches, where in most cases, the entire ranch was surveyed. To adequately survey these ranches, surveyors chose enough listening points to assure that all portions of the ranch were within 0.5 miles of a listening point. Similar to known lek surveys, the ad hoc surveys were conducted under good weather conditions, during the same time window, and during the same date range. Surveyors considered the following when choosing ad hoc survey areas: 1) the presence of suitable lesser prairie-chicken habitat, 2) areas that have never been surveyed, 3) areas that have and have not been surveyed in recent years, and 4) reports of LPC sightings. Ad hoc survey locations are recorded by GPS, buffered, and reported to Western Association of Fish and Wildlife Agencies (WAFWA).

Kansas

Observers traversed each survey route twice between March 20 and April 20 starting at 30 minutes before sunrise, listening for booming prairie chickens for 3 minutes at established stops placed at approximately 1 mile intervals. After all the listening stops had been completed, the observers backtracked along the route and flushed all the lek sites that they identified up through 90 minutes after sunrise. Observers recorded the geographic coordinates of each located lek and the total number of flushed birds. Observers were instructed to get two flush counts from each lek they identify within their standard survey area which included all habitats within

approximately 1 mile of the survey route. To get all the required flush counts, it often took additional efforts beyond the two mornings when the listening stops were completed.

New Mexico

The New Mexico Department of Game and Fish (NMDGF) conducted roadside surveys annually on 50 routes located within the known occupied and potential range of LPC. Routes were 12.8 km (8 miles) long with 9 listening points located at 1.6 km (1 mile) intervals. Each route was surveyed once between March 20 – April 30. Approximately 20 square miles were surveyed on each route totaling 1,000 square miles surveyed by this method.

Additionally, NMDGF conducted surveys on 30 Prairie Chicken Areas (PCAs) owned by the New Mexico State Game Commission. They ranged in size from 10.50 to 2,909 ha (29 to 7,189 ac) and total 27,262 acres. The entire area of each PCA was surveyed once between March 20 – April 30.

The Nature Conservancy of New Mexico conducted surveys on their 28,000 acre Milnesand Prairie Preserve near Milnesand, NM. They visited known and historic lek sights and conducted multiple counts on each active lek during March, April, and May and determined an average number of birds per lek for each lek surveyed.

Additionally, a total of 15 private ranches that are enrolled in a Candidate Conservation Agreement with Assurances (CCAA) were surveyed by the Center of Excellence (CEHMM) out of Carlsbad, NM. They visited known and historic lek sights and conducted multiple counts on each active lek during March, April, and May.

Both the Bureau of Land Management (BLM) Roswell Field Office (RFO) and Carlsbad Field Office (CFO) conducted annual surveys for LPC within their respective jurisdictions. RFO surveyed approximately 99,225 ha (245,000 ac) of LPC habitat, containing 297 known lek sites on BLM lands. The RFO visited known historic lek sites from 20 March–1 May to determine activity and bird presence. CFO conducted LPC surveys from March 6–May 15. A total of 68 listening routes, including a total 737 listening points, were surveyed for audible LPC activity. Routes were selected based on the presence of shinnery oak and/or its proximity to historical lek sites. Listening points were spaced at 0.8 km (0.5 mi) intervals, which resulted in 134,354 ha (331, 997 ac) surveyed. In addition to listening route surveys, CFO conducted surveys of historical lek sites.

Oklahoma

Oklahoma intensively ground surveyed for WAFWA range-wide conservation plan the western half of the EOR in Oklahoma in 2015 and the eastern half in 2016. These are established road-based ground survey lek routes completed twice annually from March 15-May 7 with one of the two completed in April. These mirror the WAFWA range-wide conservation plan clearance survey protocol. Approximately 25 (of 50 total) established routes were surveyed annually from 2017-2019 using the same WAFWA protocol.

Texas

All active leks on each Study Area were censused for males, females, and unknown-sex birds; in addition, lek location coordinates were taken with a GPS unit. Lek surveys generally begun during the last week of March or first week of April each year and continued until the Study Area was completely censused. Observers arrived at or near the first lek at least 30 minutes prior to sunrise. Depending on the lek, personnel made their observations from a vehicle or they walked to get as close as possible to the lek site without disturbing the birds. Observations at the first lek lasted 30-40 minutes, because males may have still been be arriving. Upon completion of observations at the first lek, observers proceeded as quickly as possible to the next site(s) because booming activity usually ceased between 8:30 and 9:00am. Data were collected and recorded on a standardized survey form.

In addition to surveying all known leks on each Study Area, a considerable amount of time was also spent in search of new leks within the Study Area. Local biologists committed up to 25% of their time during the survey period to search for new leks. When and if time and resources allowed each year and Study Area surveys were completed, TPWD field staff attempted to visit historical and/or known lek locations outside the six Study Areas and collected comparable data to those collected at Study Areas.
